# Mitochondrial DNA damage and vascular function in patients with diabetes mellitus and atherosclerotic cardiovascular disease

**DOI:** 10.1186/s12933-016-0372-y

**Published:** 2016-03-31

**Authors:** Jessica L. Fetterman, Monica Holbrook, David G. Westbrook, Jamelle A. Brown, Kyle P. Feeley, Rosa Bretón-Romero, Erika A. Linder, Brittany D. Berk, Robert M. Weisbrod, Michael E. Widlansky, Noyan Gokce, Scott W. Ballinger, Naomi M. Hamburg

**Affiliations:** Evans Department of Medicine and Whitaker Cardiovascular Institute, Boston University School of Medicine, 72 East Concord Street, E-784, Boston, MA 02118 USA; Department of Pathology, Division of Molecular and Cellular Pathology, University of Alabama at Birmingham, Birmingham, AL USA; Pharmacology and Toxicology, Medical College of Wisconsin, Milwaukee, WI USA

## Abstract

**Objective:**

Prior studies demonstrate mitochondrial dysfunction with increased reactive oxygen species generation in peripheral blood mononuclear cells in diabetes mellitus. Oxidative stress-mediated damage to mitochondrial DNA promotes atherosclerosis in animal models. Thus, we evaluated the relation of mitochondrial DNA damage in peripheral blood mononuclear cells s with vascular function in patients with diabetes mellitus and with atherosclerotic cardiovascular disease.

**Approach and results:**

We assessed non-invasive vascular function and mitochondrial DNA damage in 275 patients (age 57 ± 9 years, 60 % women) with atherosclerotic cardiovascular disease alone (N = 55), diabetes mellitus alone (N = 74), combined atherosclerotic cardiovascular disease and diabetes mellitus (N = 48), and controls age >45 without diabetes mellitus or atherosclerotic cardiovascular disease (N = 98). Mitochondrial DNA damage measured by quantitative PCR in peripheral blood mononuclear cells was higher with clinical atherosclerosis alone (0.55 ± 0.65), diabetes mellitus alone (0.65 ± 1.0), and combined clinical atherosclerosis and diabetes mellitus (0.89 ± 1.32) as compared to control subjects (0.23 ± 0.64, P < 0.0001). In multivariable models adjusting for age, sex, and relevant cardiovascular risk factors, clinical atherosclerosis and diabetes mellitus remained associated with higher mitochondrial DNA damage levels (β = 0.14 ± 0.13, P = 0.04 and β = 0.21 ± 0.13, P = 0.002, respectively). Higher mitochondrial DNA damage was associated with higher baseline pulse amplitude, a measure of arterial pulsatility, but not with flow-mediated dilation or hyperemic response, measures of vasodilator function.

**Conclusions:**

We found greater mitochondrial DNA damage in patients with diabetes mellitus and clinical atherosclerosis. The association of mitochondrial DNA damage and baseline pulse amplitude may suggest a link between mitochondrial dysfunction and excessive small artery pulsatility with potentially adverse microvascular impact.

## Background

Type 2 diabetes mellitus affects an estimated 1 in 10 Americans and this number is expected climb with the current obesity epidemic [1]. Diabetes mellitus is a significant risk factor for cardiovascular disease; however, the mechanisms behind this increased risk are incompletely understood [1, 2]. Elevated oxidant levels have been shown to contribute to vascular dysfunction both in animal models and clinical studies [3–5]. Mitochondria are an important source and target of oxidants that may contribute to vascular disease in diabetes mellitus [6–8].

Mitochondrial DNA is more susceptible to oxidative damage compared to nuclear DNA due to multiple factors including a limited repair capacity and close proximity to the electron transport chain [9, 10]. Mitochondrial DNA damage has been closely associated with dysfunctional oxidative phosphorylation, which leads to further oxidative stress resulting in a positive-feedback cycle. In an animal model of atherosclerosis, excess mitochondrial DNA damage promoted atherosclerosis and plaque vulnerability through increased monocyte activation [11]. In a prior human study of patients with coronary artery disease, the extent of mitochondrial DNA damage in circulating white cells was associated with high risk plaque burden [12]. We have previously described altered mitochondrial oxidative phosphorylation, membrane potential and morphology in peripheral blood mononuclear cells which was associated with vascular dysfunction in patients with diabetes [7, 13]. The objective of the present study was to assess the relation of mitochondrial DNA damage in peripheral blood mononuclear cells to vascular function and the presence of diabetes mellitus and atherosclerotic cardiovascular disease.

## Methods

### Study participants

We enrolled four groups of patients (N = 275): (1) clinically established atherosclerotic cardiovascular disease (Athero; coronary artery disease and/or peripheral artery disease); (2) diabetes mellitus (DM; fasting glucose levels >126 mg/dL or medication therapy); (3) diabetes mellitus and atherosclerosis (Athero + DM); (4) controls with no clinically established atherosclerosis, no diabetes mellitus (fasting glucose <100 mg/dL) and age >45 years. Patients with clinical atherosclerotic cardiovascular disease were enrolled from outpatient cardiology and vascular surgery practices. Coronary artery disease was defined based on angiography or documented history of myocardial infarction. Peripheral artery disease was defined as ankle brachial index ≤0.9 or prior peripheral revascularization. All participants gave written informed consent and all study protocols were approved by the Boston Medical Center Institutional Review Board.

### Study protocol

Clinical history and relevant clinical covariates were compiled from participant interviews and medical records. Blood pressure was assessed with an automatic recorder (Dinamap; General Electric Healthcare) and body mass index (BMI) was calculated from measured height and weight. All studies were performed in the fasted state. Peripheral blood mononuclear cells were isolated by differential centrifugation of a blood sample. Briefly, venous blood was collected into a density gradient solution for the isolation of lymphocytes and monocytes (BD Vacutainer CPT cell preparation tubes with sodium citrate; Becton, Dickinson, and Company) and the tubes were spun at 3000 rpm for 30 min at room temperature. Cell layers were collected, pelleted, and stored at −80 °C until further processing for quantitative PCR.

### Vascular function testing

Conduit arterial endothelial function was assessed using flow-mediated vasodilation. Hyperemic flow serves as the stimulus for vasodilation through endothelial-derived nitric oxide. A Toshiba SSH-140A ultrasound system was used to determine brachial artery diameter at baseline and for 1 min following 5 min of forearm cuff occlusion as previously described [14]. Brachial artery diameter was also measured prior to and 3 min following administration of 0.4 mg sublingual nitroglycerin. Nitroglycerin was not administered if the participant declined or had previous adverse reactions to nitroglycerin, migraine headaches, severe carotid stenosis, systolic blood pressure <100 mmHg, or had used sildenafil, tadalafil, or vardenafil within 1 week of their study visit. Flow-mediated dilation data was assessed with commercially available software (Brachial Analyzer version 3.2.3, Medical Imaging Applications).

### Peripheral arterial tonometry

Digital pulse amplitude was measured using a PAT device (Endo-PAT2000, Itamar Medical, Caesarea, Israel) placed on the tip of each index finger. Baseline pulse amplitude was assessed in each finger for 2 min and 20 s following which arterial flow was interrupted for 5 min with a cuff placed on a proximal forearm (Hokanson AG101, D.E. Hokanson Inc, Bellevue Was). The PAT result is expressed as the natural logarithm of the ratio of the pulse amplitude during the 90–120 s period after cuff release in the test finger to the baseline amplitude divided by the similar ratio from the contralateral control finger (ln PAT ratio) [15, 16].

### Mitochondrial DNA damage quantification

Quantitative PCR was used to measure mitochondrial DNA damage in peripheral blood mononuclear cells from patients as previously described [10, 17]. Total DNA was extracted using the QIAmp DNA mini kit (Qiagen, Hilden, Germany) and quantified using Picogreen (Invitrogen, Carlsbad, CA). A large fragment of the mitochondrial genome was amplified (16.2 kb) and normalized to copy number using a short quantitative PCR reaction (236 bp). Using this method, the polymerase remains bound to undamaged DNA and replicates a full length product; however in the presence of damaged DNA (e.g. stand breaks, cross-links, or bulky adducts), the polymerase stalls and can no longer produce full length products resulting in a decrease in amplification. The lesion frequency (LF) was calculated as previously described and is an estimate of the average number of lesions per copy of mitochondrial DNA [10, 17]. Mitochondrial DNA copy number is the quantification of the 236 bp short PCR product.

### Statistical analyses

Clinical characteristics and vascular measures were compared across the four groups (controls, atherosclerosis alone, diabetes mellitus alone, and atherosclerosis with diabetes mellitus) using one-way ANOVA or Chi squared testing for continuous or categorical data, respectively. Mitochondrial DNA damage was compared across the four groups using one-way ANOVA with post hoc testing for intergroup comparisons. Linear regression models were used to assess the association of mitochondrial DNA damage with diabetes mellitus, atherosclerosis, and cardiovascular risk factors. We performed a multivariable linear regression model comparing the relation of mitochondrial DNA damage to diabetes mellitus and atherosclerosis after adjusting for cardiovascular risk factors including age, sex, race, hypertension, BMI, smoking ever, hypercholesterolemia, and systolic blood pressure. We compared mitochondrial DNA damage and vascular measures with Spearman’s correlations. In an unadjusted model, the sample size of 275 has 80 % power to detect a correlation coefficient of 0.16 and 90 % power to detect a correlation coefficient of 0.19. Hence, the study has sufficient power to evaluate for a modest correlation coefficient between vascular measures and clinical characteristics. Data are reported as mean ± SD, unless otherwise specified. Two-sided P < 0.05 was considered statistically significant. All statistical analyses were carried out using IBM SPSS Statistics version 20.

## Results

### Study population

The clinical characteristics and vascular measures across the four groups are summarized in Table 1. The overall mean age was 57 ± 9 years with 60 % female and 38 % black race. Fewer women were represented in the atherosclerosis (CAD and/or PAD) group than men. As expected, atherosclerosis alone or in the presence of diabetes mellitus was associated with higher burden of cardiovascular risk factors. Additionally, the prevalence of cardiometabolic factors was higher in the diabetes mellitus alone group. Consistent with prior reports, multiple measures of vascular function were impaired with atherosclerosis, diabetes mellitus, and atherosclerosis and diabetes mellitus.

**Table 1:** Clinical characteristics

	No athero or DM (N = 98)	Athero alone (N = 55)	DM alone (N = 74)	Athero and DM (N = 48)
Clinical characteristics				
Age, years	55 ± 7	60 ± 10	55 ± 10	62 ± 8
Female, %	49	18	47	31
Black race, %	31	27	65	29
Hypertension, %	21	67*	70*	88*
Hypercholesterolemia, %	56	82*	61*	85*
Smoking ever, %	47	78	58	64
Family history of CAD, %	32	38	27	46
Body mass index, kg/m^2^	27.6 ± 5.0	29.6 ± 6.0*	34.3 ± 8.4*	31.3 ± 4.9*
Systolic BP, mmHg	123 ± 15	130 ± 20*	135 ± 18*	141 ± 20 *
Diastolic BP, mmHg	73 ± 9	74 ± 11	76 ± 8	78 ± 27
Total cholesterol, mg/dl	204 ± 42	168 ± 39*	176 ± 37*	150 ± 39*
LDL cholesterol, mg/dl	124 ± 40	97 ± 31*	101 ± 29*	82 ± 32*
HDL cholesterol, mg/dl	58 ± 22	45 ± 14*	46 ± 15*	42 ± 13*
Triglycerides, mg/dl	112 ± 82	134 ± 76	147 ± 95*	135 ± 95
Fasting glucose, mg/dl	92 ± 12	107 ± 27*	181 ± 85*	163 ± 73*
Insulin, uU/ml	7.4 ± 5.6	13.3 ± 13.8*	17.7 ± 25.6*	13.2 ± 11.5*
ACE inhibitor or ARB therapy, N (%)	8	45*	46*	54*
β-blocker therapy, N (%)	7	44*	23*	62*
Lipid lowering therapy, N (%)	9	87*	42*	71*
Antiplatelet therapy, N (%)	15	93*	44*	69*
Insulin therapy, N (%)	0	0	43*	38*
Metformin therapy, N (%)	0	0	53*	33*
Sulfonylureas, N (%)	0	0	7*	8*
Vascular measures				
Baseline brachial diameter, mm	4.11 ± 0.65	4.57 ± 0.63*	4.39 ± 0.75	4.46 ± 0.64*
Baseline brachial velocity, cm/s	16 ± 9	16 ± 6	16 ± 6	18 ± 8
Brachial artery flow-mediated dilation, %	9.3 ± 4.5	7.0 ± 3.9*	6.6 ± 4.3*	6.8 ± 4.0*
Nitroglycerin-mediated dilation, %	13.5 ± 6.1	9.2 ± 5.7*	10.4 ± 5.1	9.2 ± 6.1*
ln baseline pulse amplitude	5.7 ± 0.88	6.4 ± 0.63*	6.0 ± 0.78	6.3 ± 0.73*
ln PAT ratio	0.75 ± 0.37	0.56 ± 0.33*	0.64 ± 0.39	0.55 ± 0.51

Mean ± standard deviation or percent as appropriate

*Athero* atherosclerosis, *DM* diabetes mellitus, *CAD* coronary artery disease, *BP* blood pressure, *LDL* low density lipoprotein, *HDL* high density lipoprotein, *ACE* angiotensin-converting enzyme, *ARB* angiotensin receptor blocker, *PAT* peripheral artery tonometry

* P < 0.01 by ANOVA or Chi squared as appropriate

### Association of mitochondrial DNA damage with diabetes mellitus and atherosclerosis

**Fig. 1:**
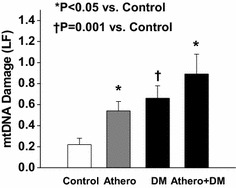
Mitochondrial DNA damage was higher in in the presence of DM alone, athero alone, and athero + DM as compared to controls (*P < 0.05 vs control, †P = 0.001 vs control)

Mitochondrial DNA damage was higher in each of the 3 patient groups (atherosclerosis alone, diabetes mellitus alone, and combined atherosclerosis and diabetes mellitus) as compared to controls (Fig. 1). We did not observe any differences in mitochondrial DNA copy number between the patient groups (atherosclerosis alone, 0.76 ± 0.73; diabetes mellitus alone, 0.76 ± 0.45; atherosclerosis and diabetes mellitus, 1.2 ± 1.0) compared to controls (1.0 ± 1.08, P = NS). As shown in Table 2, diabetes mellitus and atherosclerosis were associated with higher mitochondrial DNA damage in unadjusted models and in models adjusting for the presence of cardiovascular risk factors. Additional cardiovascular risk factors were not associated with the extent of mitochondrial DNA damage. Mitochondrial DNA damage correlated with measures of fasting glucose (r = 0.130, P = 0.037) and HOMA-IR (r = 0.155, P = 0.02) across the entire study group. However, the association of mitochondrial DNA damage with fasting glucose (r = −0.16, P = 0.869) and HOMA-IR (r = 0.091, P = 0.353) was not significant in the subgroup of patients with diabetes mellitus suggesting that the presence of diabetes is the important driver of the association. Mitochondrial DNA damage remained associated with diabetes mellitus and atherosclerosis in models including cardiovascular and antidiabetic therapies (ACE inhibitor or ARB therapy, β-blocker therapy, lipid lowering therapy, antiplatelet therapy, insulin therapy, metformin therapy, or sulfonylureas) as potential covariates.

**Table 2:** Relations between mitochondrial DNA damage and vascular risk factors

	Mitochondrial DNA damage			
	Bivariate		Multivariable	
	β (SE)	P	β (SE)	P
Diabetes mellitus	0.220 (0.110)	<0.001	0.213 (0.127)	0.002
Atherosclerosis	0.153 (0.115)	0.01	0.140 (0.134)	0.04
Hypertension	0.107 (0.112)	0.08	−0.037 (0.137)	0.6
Hypercholesterolemia	0.136 (0.120)	0.02	0.093 (0.124)	0.1
Smoking ever	0.034 (0.115)	0.6	−0.007 (0.117)	0.9
Age, years	0.003 (0.006)	0.6	−0.030 (0.007)	0.6
Sex	0.046 (0.115)	0.4	0.023 (0.120)	0.7
Race	0.012 (0.115)	0.842	0.002 (0.124)	0.9
BMI	0.099 (0.008)	0.1	0.043 (0.009)	0.5

### Comparison of mitochondrial DNA damage with measures of vascular function

As shown in Table 3, there was an association of higher mitochondrial DNA damage with higher baseline pulse amplitude (P = 0.009). Other measures of vascular function including baseline brachial diameter, flow-mediated vasodilation, nitroglycerin-mediated vasodilation, and PAT ratio were not associated with mitochondrial DNA damage. In a multivariable model adjusting for cardiovascular risk factors (age, sex, body-mass index, smoking, hypertension, and diabetes) mitochondrial DNA damage remained associated with higher baseline pulse amplitude (β = 67 ± 31, P = 0.03).

**Table 3:** Relation of mitochondrial DNA damage and vascular function

	Mitochondrial DNA damage	
	r	P
Baseline brachial diameter, mm	0.114	0.06
Flow-mediated dilation, %	−0.026	0.7
Nitroglycerin-mediated dilation, %	−0.02	0.8
Baseline pulse amplitude, au	0.19	0.009
ln PAT ratio	−0.086	0.2

*PAT* peripheral arterial tonometry

## Discussion

In this study, we evaluated mitochondrial DNA damage in peripheral blood mononuclear cells and related the findings to vascular function in a large group of patients with diabetes mellitus, clinical atherosclerosis, and controls. We observed higher mitochondrial DNA damage in peripheral blood mononuclear cells in association with both diabetes mellitus and clinical atherosclerotic disease when accounting for concomitant cardiovascular risk factors including age. We observed similar levels of mitochondrial DNA damage in patients with diabetes mellitus compared to established atherosclerotic disease, which is consistent with the concept of diabetes as a coronary disease equivalent. No differences in mitochondrial DNA copy number were observed in any of the patient groups relative to the controls suggesting that the differences in mitochondrial DNA damage were not due to alterations in mitochondrial DNA copy number. Mitochondrial DNA damage was associated with higher baseline pulse amplitude consistent with an alteration of microvascular pulsatility. In contrast, there were no associations of mitochondrial DNA damage with measures of vasodilator responses in either conduit or microvessels. Taken together, our findings provide evidence that diabetes mellitus and atherosclerotic cardiovascular disease are associated with increased mitochondrial DNA damage that may be relevant to oxidative stress and microvascular dysfunction.

Prior work indicates a link between mitochondrial alterations, diabetes mellitus and atherosclerosis. In previous work by our group, we found that peripheral blood mononuclear cells from patients with diabetes mellitus had an abnormal mitochondrial phenotype characterized by higher mitochondrial oxidant production, mitochondrial membrane hyperpolarization, higher uncoupled respiration, and fragmented mitochondria [7, 13]. In the current study, we have extended our previous work by measuring mitochondrial DNA damage in circulating white cells. Mitochondrial DNA damage is postulated as a measure of environmental injury to the mitochondria that may mediate mitochondrial dysfunction. The ability to measure mitochondrial DNA damage in circulating white cells has potential utility as a biomarker of mitochondrial injury. Damage to mitochondrial DNA has been shown to mediate mitochondrial dysfunction including alterations in calcium handling, oxidant-mediated activation of cell stress pathways, and metabolic disturbances [5, 11, 12, 18]. Oxidative stress has been shown to induce mitochondrial DNA damage in vascular cells (endothelial and vascular smooth muscle cells) [19]. Additionally, mitochondrial DNA damage has been shown to be the result of replication errors and repair insufficiencies with the damaged mitochondrial DNA undergoing clonal expansion [20, 21]. Animal models with a genetic predisposition to increased mitochondrial DNA damage display mitochondrial dysfunction and accelerated atherosclerosis [11, 18]. Interestingly, enhanced mitochondrial DNA damage was associated with abnormal metabolic function including glucose intolerance consistent with a connection to diabetes mellitus [16]. Further, monocytes with increased mitochondrial DNA damage were pro-inflammatory and were associated with increased plaque vulnerability [17].

Several prior studies have measured specific mitochondrial DNA changes in blood cells from patients with atherosclerosis and diabetes mellitus. Patients with coronary artery disease were found to have higher levels of the common mitochondrial DNA 4977 bp deletion in circulating peripheral blood mononuclear cells compared to age matched, healthy controls [22]. In a study of patients with coronary artery disease, higher mitochondrial DNA damage was associated with vulnerable plaque characteristics and history of diabetes mellitus [18]. In an additional study, peripheral blood cells from diabetic patients had higher levels of a specific mitochondrial DNA point mutation (A3243G) compared to non-diabetic controls [23]. Using a sensitive quantitative PCR based technique to determine mitochondrial DNA damage, we demonstrate the presence of higher mitochondrial DNA damage in peripheral blood mononuclear cells both in patients with diabetes mellitus and with clinical atherosclerosis [10, 17]. This method allowed us to assess damage throughout the mitochondrial genome rather than at a single nucleotide position and is sensitive to strand-breaks, deletions, dimers, cross-linking, and bulky adducts allowing us to assess a number of different types of damage. Our findings suggest the independent association of both metabolic disease and atherosclerosis with mitochondrial DNA damage even when accounting for associated cardiovascular risk factors. The greater extent of mitochondrial DNA damage may drive increased production of mitochondrial reactive oxygen species and accentuate vascular damage in diabetes mellitus. The association of mitochondrial DNA damage with atherosclerosis suggests that mitochondrial DNA damage measures in a blood sample may serve as novel biomarkers of cardiovascular injury. Future studies are needed to evaluate the predictive value of mitochondrial DNA damage for cardiovascular events in a longitudinal study.

Several lines of evidence indicate the relevance of mitochondrial phenotype to vascular function [24]. Mitochondrial dysfunction in the endothelium disrupts nitric oxide signaling and impairs vasodilation [6, 25]. Relevant to the present work, studies of peripheral blood mononuclear cells have shown an association of lower mitochondrial mass, higher mitochondrial superoxide production, and altered oxidative phosphorylation with impaired vascular function in diabetes mellitus [7, 13]. We now show that mitochondrial DNA damage relates to higher baseline pulse amplitude measured in the fingertip circulation. Baseline pulse amplitude measures microvessel pulsatility and is governed by blood flow, vessel compliance and sympathetic tone. Higher small vessel pulsatility may lead to microcirculatory injury and has been associated with several cardiovascular risk factors [15, 26]. In one prior study of high risk patients, baseline pulse amplitude was not associated with large vessel cardiovascular events; however, the predictive value for microcirculatory events is not known [27]. The relation of mitochondrial DNA damage to higher pulse amplitude may reflect an impact on resting sympathetic tone or small vessel compliance.

In contrast, we did not observe associations of mitochondrial DNA damage with measures conduit and microvascular vasodilator function. The present finding differs from our prior studies showing impaired flow-mediated dilation and peripheral arterial tonometry hyperemic response in the presence of mitochondrial dysfunction. The lack of association may suggest that mitochondrial DNA damage is not an important mediator of endothelial dysfunction. It is also possible that mitochondrial DNA damage does not directly modulate mitochondrial reactive oxygen species generation in peripheral blood mononuclear cells. A recent study demonstrated that a novel regulator of kinases and phosphatases, prolyl-isomerase-1 (Pin1), mediates alterations in mitochondrial oxidant production and downstream vascular signaling pathways relevant to vascular tone and inflammation [28]. Pin1 levels were higher in the presence of diabetes and associated with impaired endothelial function and higher oxidative stress biomarkers, consistent with a connection between mitochondrial DNA damage and vascular function in patients [28]. Hence, it remains possible that greater mitochondrial DNA damage extent or injury in vascular cells would lead to vasodilator dysfunction. Additional studies will be necessary to define whether the microvascular changes observed with mitochondrial DNA damage in peripheral blood mononuclear cells contribute to target-organ consequences and the relevance of mitochondrial DNA damage in cells types important to vascular disease including leukocytes, platelets, endothelial cells, and smooth muscle cells.

Our study has a number of limitations. We assessed mitochondrial DNA damage in peripheral blood mononuclear cells and not a vascular tissue such as endothelial cells or vascular smooth muscle cells from patients. However, peripheral blood mononuclear cells are a readily accessible cell type in human subjects and are exposed to the same systemic risk factors. Additionally, due to the study design, we are limited to identifying correlations of mitochondrial DNA damage with vascular function rather than causal relations. Some of the types of mitochondrial DNA damage measured using the quantitative PCR technique may not affect mitochondrial function. However, we did observe an association of mitochondrial DNA damage with atherosclerosis and diabetes mellitus. One of the reasons for a lack of association of several vascular function measures with mitochondrial DNA damage may be due to the global assessment of mitochondrial DNA damage rather than measuring damage or mutation of a specific site of mitochondrial DNA. Further, mitochondria are responsible for multiple cellular activities including calcium storage, apoptosis, metabolism, regulation of cell growth, and heme biosynthesis. The optimal assessment of mitochondrial function is likely to combine multiple facets including mitochondrial oxidative stress, mitochondrial structure, mitochondrial complex activity, and mitochondrial DNA damage. Future work will be needed to assess the relationship of the multiple facets of mitochondrial function with vascular measures. We found an association of mitochondrial DNA damage with the presence of clinically evident atherosclerosis; however, we do not have measurements of subclinical atherosclerosis such as carotid-intima media thickness in our patient sample. Finally, further studies will be needed to define the mechanisms linking mitochondrial DNA damage to risk factors and to microvascular alterations. Strengths include the relatively large sample size, the comprehensive noninvasive assessment of vascular function, and the use of established quantitative PCR based methodology to measure mitochondrial DNA damage.

## Conclusions

Our main findings indicate an association of mitochondrial DNA damage in peripheral blood mononuclear cells with microvascular pulsatility but not vasodilator function. Furthermore, both diabetes mellitus and clinical atherosclerotic disease associate with greater mitochondrial DNA damage consistent with prior work showing abnormalities in mitochondrial function. Additional work is required to elucidate the contribution of mitochondrial phenotype in microvascular disease and whether therapies aimed at protecting the mitochondria from DNA damage will have cardiovascular benefit.
